# Making virtual learning engaging and interactive

**DOI:** 10.1096/fba.2020-00084

**Published:** 2020-12-05

**Authors:** David S. Sweetman

**Affiliations:** ^1^ University of Michigan Ann Arbor MI USA

**Keywords:** asynchronous and synchronous learning, online learning, small group learning, social interaction, virtual classes, zoom

## Abstract

This article provides practical perspective and guidance for transitioning from in‐person to virtual learning. Student engagement is emphasized through providing synchronous and highly interactive virtual learning sessions. This approach not only improves student outcomes related to class, but also is related to strong student mental health. Technological considerations are explored surrounding how to select a video conferencing platform that will enable engagement. Key functionality recommended includes the ability to share audio and video of both instructor and students, virtual hand raising and other signaling capabilities, hosting of small group discussions within the larger class, concurrent chat capabilities, and the crowd‐sourcing of questions. The implications of this functionality from a learning perspective are discussed. Empathy and flexibility in accommodating diverse and evolving student scenarios is also discussed. The importance of setting norms and expectations provides a foundation for the class, both during class sessions as well as in providing a framework within which students conceptualize group work. This article ends by looking ahead at near‐term implications of teaching during a global pandemic.

## 1 INTRODUCTION

In a recent analysis of successful life science research exemplars, all of the most important characteristics identified by these exemplars related to the human dimension of research: relationships, passion, and resilience were the top three characteristics.^1^ A key part of the learning experience of these exemplars began in the same place it does for all of us: in the classroom. Therefore, teaching the human dimension of work is an important part of our pedagogy. This is best made possible through interactive learning experiences.^2^

As a faculty member at a large, resident‐based public university, I have always placed value on the importance of in‐person education. A key part of that value has been in the interaction. Interaction between students and professor, and interaction between students. This is true whether a small seminar class of 15, a large survey course of 150, or anywhere in‐between. Last spring as we began life with the COVID‐19 pandemic, I was suddenly forced from the familiarity of teaching those engaging classes in‐person to transitioning to an all remote interaction. That semester, I was teaching a 150 person introductory course on organizational leadership and a 30 person capstone seminar course on strategic change through human resource management. Both were taught in‐person for the first half of the semester before transitioning, and both were undergraduate courses, with the introductory course being largely sophomores and juniors while the seminar course was largely seniors. Additionally, at the end of the term, I virtually hosted a traditionally 3‐day in‐person summer program for over 250 high school students. Like so many peers around the world, I feverishly worked to transition those well‐planned in‐person learning interactions to a format where I found myself physically in a room by myself. It took roughly one hour for every hour of class time to thoughtfully plan and update materials to transition the course from in‐person to virtual formats. While this experience is not what I planned, nor what I thought I would have wanted, I went all‐in to make the best of the situation. As expressed in end‐of‐semester evaluations, a number of students actually found the virtual experience *more* engaging than in‐person class. What a pleasant surprise. I too had found unexpected positives in the transition to virtual interaction. In this article, I share my perspectives and experiences of what worked (and what didn’t) and end with specific recommendations for you to consider when moving forward with your own online teaching. This article focuses specifically on the experience of interpersonal interaction within the classroom, especially in fostering interactive dialog among learners. The present article does not address tools specific to STEM education, such as interactive anatomy tools, which are covered elsewhere in this special issue.^3^

## 2 AGAINST COMMON WISDOM: SYNCHRONOUS LEARNING

The first major decision I faced: do I continue to offer the course at the same scheduled time and expect students to attend at that time (synchronous), or do I simply record lectures and structure assignments such that there is the flexibility of viewing and completing coursework when convenient (asynchronous)? Guidance and resources emerged and the common wisdom I largely saw was to go asynchronous—to video record lectures and perhaps offer chat board interaction via the institution’s Learning Management System. However, I chose not to do that. I chose to go the synchronous route, because the learning of the subject matter I teach is maximized through synchronous discussion. Students retain and apply materials better through the process of discussion.^4^ When my university ran usage data on video conferencing a few weeks into the virtual transition, I in fact found my magnitude of adopting synchronous learning was among the highest in the university—one of the top five greatest volumes of usage of almost 13,000 users of the video conferencing platform at the university.

Additionally, as we consider the undergraduate residential college experience, it is largely defined by a regular sense of structure, and regular social interaction with peers. This has been well‐established in decades of meta‐analyses on achievement in higher education.^5^ The cancelling of in‐person learning and extracurricular experiences served to take away both of those pillars of the educational experience. While our collective concerns and attention were largely toward the physical health of students, I was already considering the mental health impacts of an abrupt transition from the regular structure and interactions of college life to being relatively isolated with relatively little structure to individual schedules. Recent survey data has shown a 40% increase rates of mental health impairing academic performance when compared to pre‐pandemic levels.^6^ This underscores the importance of efforts to keep students engaged and mentally healthy.

The argument in favor of synchronous learning is in some ways parallel to the argument for active learning and “flipping the classroom” in biomedical education. “Flipping the classroom” refers to providing pre‐reads and recorded lectures ahead of class time so that class time is focused on interactive pedagogy to solidify and synthesize student learning. Systematic review has found inconclusive results in “flipping the classroom.”^7^ However, there is an important distinction to be made. Increased outcomes are not always associated with a “flipped” delivery per se, but rather through the active learning that is often enabled in this approach.^8^ The considerations related to synchronous learning discussed in this paper relate to enabling an active learning approach, which may or may not include a “flipped classroom” instructional design. Additionally, synchronous classroom experience helps ensure appropriate pacing of the delivery of materials, rather than students binging multiple lectures in a short period of time. While some students may desire the autonomy of asynchronous learning, the benefits of active learning can best be realized by both undergraduate and graduate student populations through actively engaged synchronous learning experiences.^9^

I’m glad I made the decision to maintain synchronous learning. Throughout the semester, I received *unsolicited* positive feedback from over 75% of students for that decision. This feedback took two main forms. First, from the perspective of educational experience, students had been worried. They had been worried they would “lose out” or otherwise have a “subpar” learning experience due to the transition. As the semester went on, this feedback became even more pointed in comparison with other classes they had that had gone asynchronous, and how the video recordings and asynchronous discussion boards simply weren’t providing them a meaningful level of engagement and learning. As an example of the feedback: “Thank you for not letting the class experience be lost just because we could no longer meet in‐person. I still learned so much meeting together on Zoom.” Synchronous class facilitated both engagement with the material as well as their peers to produce a high‐quality learning experience.^10^

The second form of positive feedback from students on synchronous classes was very personal. I was moved by the number of students who told me my class was something that they looked forward to each week, to a much greater degree than when it was in‐person. Some went as far as to call it the highlight of each week and something that helped them to stay mentally healthy. This excitement for the class was grounded in it being one of the only regular times they had the opportunity for real, meaningful interaction with people outside of their immediate household.

To be clear, both in‐person and virtually, class is structured in a highly interactive form. I strive to limit my guidance through course materials to no more than 10 minutes at a time, and less than half of the total class time. The balance of class time is spent in large‐group discussion, small group breakout discussions, or other activities designed to apply and solidify the learning objectives for the day’s class. While I did not fundamentally change this interactive approach from in‐person to remote learning, I realize that perhaps student attention spans are shorter and more readily distractible when attending virtually rather than in‐person. As such, the focus on a highly interactive and engaging experience is even more important to the success of the virtual class setting.

## 3 TEACHING GLOBALLY ACROSS DIFFERENT TIME ZONES

A factor that could have limited my decision to teach synchronously was that I teach to a global audience. Students had returned to their homes and were now scattered across ten time zones throughout the world. Fortunately, the timing of the classes and the time differences largely worked. I teach in the Eastern time zone, and my only complaints came for my 10am class from a few students on the west coast of the United States, where class started at 7am for them. While perhaps not ideal for their learning experience, I held firm that this was still doable and expected.

On the other extreme, the other course I taught was an evening course that ended at 7pm, which meant 1am for a few students joining from Europe. While they were used to late‐nights anyways, their parents were not. So, while these students joined the class, their vocal contributions to discussions ended mid‐way through class once their parents went to bed for the night so as to not disturb them. As we look ahead to continued online teaching experiences, factoring in student time zones of residence should be a key component of scheduling synchronous learning experiences. We need to know time zones where students will be living and find a time that best accommodates the greatest number of learners.

## 4 TECHNOLOGY PLATFORM CONSIDERATIONS

I reviewed and experimented with a number of different video conferencing platforms for the transition to online education. Some of the largest platforms in the market include Adobe Connect, BlueJeans, Cisco WebEx, Google Hangouts, Microsoft Teams, and Zoom. Check with your institution’s information technology group, as many have purchased a site license for one or more products and integrated it with other student technology. One of the most important factors in deciding what platform to use is what platform(s) are licensed and supported by your institution, which also includes the benefit of student familiarity and integration with other tools, such as the Learning Management System. Additionally, as you consider which platform(s) to use, it is important to have clear requirements in mind of what you do, and don’t, need a virtual platform to do to achieve your pedagogical goals. For me, factors that were especially important were: ability to share video and audio, virtual hand raising, small group discussions, chat, crowd‐sourced questions. The software I ended up using to accomplish all of this was Zoom. This section is neither an advertisement for or against any particular video conferencing platform, but I use the example of Zoom as it is something that worked for me.

### 4.1 Audio and video

This is standard fare for any video conferencing platform. However, of specific interest to me was the ability to see many students all at once. When we teach in‐person, consciously or not, we will often “read the room” of student non‐verbal reactions to understand student engagement, the degree to which they understand materials, or their general energy level. We then adapt to the needs of the students to maximize their learning. Being able to see students during class was therefore an important piece for me of any technology tool.

While not related to the technology specifically, this also implies the expectation that students have their video turned on and shared so you and others in the class can see them. Meta‐analysis has shown this shared social presence to positively impact learning outcomes.^11^ It is important to establish this standard expectation early in the semester. Otherwise, you run the risk of it becoming the habit of students to not turn on their video, and that habit is much harder to break later. That said, I do not recommend requiring video be turned on, but only strongly encouraging it. Depending on student circumstances, living environment, and Internet availability, not all students may be able to share video.

As for audio, to minimize distraction and maximize focus on learning, students should only turn on their audio when they are talking, and mute their audio when they are not. This avoids background noises and the annoying audio feedback that can sometimes plague video conferencing calls. I found I would need to occasionally remind a student to unmute themselves so the class can hear them, but students are largely used to the technology and self‐regulating their muting and unmuting.

### 4.2 Screen sharing

This is also standard fare for any video conferencing platform. The ability to share all or a portion of one’s screen can be used for presentations and other materials. Students have the flexibility to view both the presentation and instructor when the screen is shared. Additionally, sometimes sharing materials on screen and sometimes focusing on video sharing is a good way to provide variety for students during the presentation.

Instructors and students both have the option to share their screen. This is configurable in most video platforms so that you can choose to enable or disable it. However, I found it presented flexibility and another tool in our toolkit to use to achieve our learning goals. While student‐led presentations are perhaps an obvious example of where this was useful, a less‐obvious example is as a way to visually share output or summary from small group discussions, or for students to be able to showcase learning objects they have uncovered that other students may find useful.

### 4.3 Virtual hand raising

Again compared to the in‐person class experience, something as simple as raising one’s hand needs thoughtful translation to a digital context. While physically raising one’s hand in a video conference can work for smaller class sizes, it becomes more difficult to see that in larger classes, not to mention not all students can participate via video. The virtual platform needed to include the equivalent of hand raising, where students click a “raise hand” button and the instructor and peers see a visual indicator.

Unexpectedly, this is one of the areas I found worked out better virtually than the in‐person classroom. There were two reasons for this. First of all, there was no mistaking whether or not the person was raising their hand. In‐person, there can be the timid hand raise that barely looks like a hand raise, or even the adjusting of hair that can be mistaken for a hand raise. However, with the software, the indicator is clear: either the hand is raised or not. Additionally, the software keeps track of the order in which hands are raised and students were listed to me in that order. While hand raising was a key feature to facilitating large‐group discussion, a solution is also needed for small group discussion.

### 4.4 Small group discussions

This is similar to the idea of having small tables in a physical classroom and asking students to have a discussion at their small tables. This was perhaps the feature that made the most difference to the learning experience, as it is a learning tool I use regularly in the physical classroom, especially to benefit conceptual learning.^12^ This functionality put students into separate groups, each of which was an independent discussion. Both myself and teaching team members could each then “visit” those individual discussion groups, much like walking around an in‐person classroom and talking with each of the groups. Students can also effectively “raise their hand” to signal if they’d like the attention of a member of the teaching team.

The small group discussions functionality also had great flexibility—I chose the number of students per group and could pre‐assign or randomly assign specific students to specific groups. Especially for my larger class of 150, this was even better than the physical classroom as I could readily configure differing discussion group sizes with differing amounts of people in a way that would have been impossible—or taken up huge amounts of time to logistically move students ‐ in a lecture hall of 150 students. For example, in the course of one 90 minute class session, I had students begin the class session by connecting in a small group, mimicking time for chit‐chat when students sit at the same table together before class begins. At two later points in the session, I had them in breakout groups of seven, each one of these groups having different members. Finally, I had a couple quick back‐to‐back small groups of three, each with different peers. I would have never tried to accomplish that many changes in the course of one in‐person class session.

Additionally, as each breakout room was independent, small group discussions did not have to deal with the background noise of other groups talking at the same time. However, with that great benefit came a great limitation as an instructor: it was not possible to “read the room” to decide whether to extend or reduce the amount of discussion time. When multiple small groups are talking concurrently in a large room, you can sense the level and intensity of discussion and whether to continue or cut short. However, this insight is not possible in the virtual small group discussions. As such, I had to be much more intentional in planning discussion content and the amount of time it would take.

### 4.5 Chat

When I started on this journey, I did not expect to use the chat function much, however, it filled an additive role to the overall experience. On a simple level, if I had a question that quickly elicited short answers from many people, chat was the best tool. For example, when I’m looking for many people to provide examples of a specific phenomenon. On a more complex level, it supplemented discussion and in some ways took the place of “sidebar conversations” students may have had one‐on‐one during a discussion, except now those conversations were with the entire class. For example, students would sometimes add sub‐points or personal examples while another student was talking in order to facilitate peer learning.^13^ Additionally, after individual or group presentations by students, chat was a way peers could affirm each other’s presenting or ask follow‐up questions.

Chat can also be implemented privately with a given student. Chat made it easy for me to converse with students individually if needed during class. This was sometimes used for technical issues, such as helping a student who was having microphone difficulties. Related to learning, individual chat also provides an opportunity to address students individually. For example, if I noticed a student who looked puzzled on video, or made a discussion comment that showed they weren’t quite understanding the material, I could offer a personalized follow‐up.

### 4.6 Crowd‐sourced questions

I will routinely host guest speakers during the semester. When I do, I provide students the opportunity to ask questions of the guests. I’ve often had concerns with that format, wondering if the students who were speaking were asking questions representative of the class as a whole, or relatively more unique and one‐off to their personal interests. That concern has now disappeared for me in the virtual format. In this format, students have the ability to pose a question for fellow students to see it and then “vote up” questions that also interest them. Within a few short minutes of the start of the session, I quickly had a list of questions organized by the number of students who were interested in each question being asked. Students also experience an increased sense of engagement through working collaboratively to determine questions for guests. This functionality is something I plan to introduce back into my in‐person classes, as standalone functionality like this is also available through tools such as Slido.

While it could have been simple for me to then ask those questions of guest speakers, I took different approaches to more directly engage students and speakers. In the case of the smaller class of 30, I would invite the person who originally posed the vote‐up question to then ask the question to the speaker. In the case of the larger class of 150, I asked for a few volunteer student moderators who would take turns asking the questions on behalf of the larger class. In both these scenarios, I also made clear the expectation and norm that students could then ask follow‐up questions if so inclined based on the speaker’s response.

While I highlight the use of this feature as related to guest speakers, one can envision other uses. For example, to elicit questions about readings or the day’s lecture to help guide discussion, or to crowd‐source examples or idea generation related to a concept being studied. Additionally, these tools can be configured such that students can respond to other student questions. In this way, there are two related discussions occurring simultaneously in class: the one where students are answering the questions of peers, and the other where you are discussing the larger and more complex questions as a class.

### 4.7 Annotation

This is a functionality of Zoom I had initially disabled, but later came to appreciate its value. Annotation provides students the ability to write on slides you are presenting. Like it was for me, that may be an idea that initially causes you some hesitation. However, it proved to be another source of interaction that is not readily possible in the in‐person classroom. Specifically, Zoom annotation provides a tool called “Stamps” where students can place a predefined stamp (for example, “X”, “O”, etc) somewhere on the screen. Interactive slides can then be crafted where students would put their stamp somewhere on the screen. For example, providing a continuum and asking students to denote where they are in understanding a topic, or providing a handful of options on the screen and asking each student to select one by putting their stamp in the appropriate place. This enables sudents to quickly see where they stand relative to their peers for a given discussion prompt.

Table 1 summarizes all the key features of video conferencing platforms. All of these technology tools that have the potential to enhance the student virtual learning experience. However, we must also remember that not all students may have access to the underlying technology or environment needed to leverage these tools for the benefit of learning. Flexibility is needed as we consider implementation.

**TABLE 1:** Key features of a video conferencing platforms

Audio and video sharing for instructor and students Screen sharing Virtual hand raising Small group discussion breakouts Chat Crowd‐sourced questions Annotation

## 5 FLEXIBILITY AND EQUITABLE ACCESS

A strong argument *against* the synchronous virtual learning discussed in this article is that it could exclude students from participating due to socioeconomic or other reasons. The technology and Internet connectivity needed for video conferencing may not be something all students have access to in their homes, and could disadvantage those who do not have that access. As such, the approach needs to be flexible to ensure equitable access. For example, I set the expectation that sharing your video was strongly encouraged, but was not required. For any given class session, a small percentage of the class would not share video at all, and their grade was not adversely affected.

In the event that a student does not have the necessary technology tools to fully participate in virtual classes, what are the options? Fortunately, this is not an issue for us to solve individually as professors. Most institutions are grappling with this question and many have limited programs available to provide assistance to students who may need it.^14^ Be aware of your institution’s approach, including knowing how to refer students who may benefit from such a program.

In addition to potential socioeconomic differences for students, we are living in an especially uncertain time. We need to be empathetic and flexible in providing asynchronous learning options when life events occur that make a particular synchronous learning experience not possible. For example, a student who is in quarantine, a student who is caring for infected relatives, or a student who has tested positive for COVID‐19 each require different forms of flexibility.

## 6 INVITED INTO STUDENT HOMES

As a professor, it is not expected or encouraged that we would go to a student’s home. In decades of teaching, I have never once set foot in a student’s home, nor has one of my students set foot in my home. Yet in a virtual environment, week after week we are invited into each other's homes during our shared learning experience. I believe this has led to a learning experience that feels much more personally connected for students. Photos, paintings, collections, bold colors, neutral colors, or even features like a back‐of‐the‐door basketball hoop, virtual background choice, or choice of headphones bring a level of seeing personality we don’t experience in the classroom. Kitchen tables, living rooms, bedrooms, basements, closets, garages, and even bathrooms and cars are all places students have joined from for my classes. With each of those, the key is to remind students that learning best occurs in a distraction‐free environment, and to ask them to thoughtfully select a location that will minimize the chance for distractions during class time. I respect that location is different for each student depending on their individual circumstances.

However, despite their best attempts at a distraction‐free location, the realities of their living environment sometimes came out during class: the 4‐year‐old niece excitedly barging into the room during a student’s final presentation, the thunderstorm that disrupted wireless Internet in the middle of class, or the loud party at the neighbor’s that could be clearly heard every time the student unmuted to speak. Sharing these virtual experiences week after week gave students a level of insight and appreciation into each other that seemed to help them build community in a way greater than a shared in‐person classroom experience. However, we also need to expect the unexpected and be ready to respond by facilitating classroom discussion with flexibility in focusing students on our learning objectives for the class.

## 7 MY TECHNOLOGY FOR TEACHING

This section provides just a brief mention of my own use of technology during class, and what I would recommend to others as a result. First, multiple computer monitors worked extremely well for me. This could be either one computer with two monitors, or two different computers. This enabled me to arrange video, chat, class list, slides, and all other materials in a way I could easily see them all. However, the more spread out your screen, the more important it is to remember where the video camera is on your device, and where you are looking relative to the camera.

It is relatively easy to look directly at students in an in‐person classroom. However, if you are looking at students on your screen, it will not appear to the students as though you are looking at them. Rather, know where the camera is on your device, and know students will experience you looking at them when you look at the camera. I found it highly unnatural to look directly at students in the virtual setting (which meant looking directly at the camera). However, as the weeks went on, it is something I became more comfortable in doing. Also, if you are working with others in the delivery of the class, be sure to have some form of communication, such as text message or group chat, that you can use to keep in contact with each other during a class session to exchange logistical and other time‐sensitive messages. This separate channel of communication ensures communication isn’t missed among student communication and keeps communication open in the event of technical issues where the connection to Zoom is lost by any member of the instructional team. Finally, private chat in Zoom is only between two individuals, so where a teaching team consists of three or more people, Zoom isn’t even an option.

**FIGURE 1:**
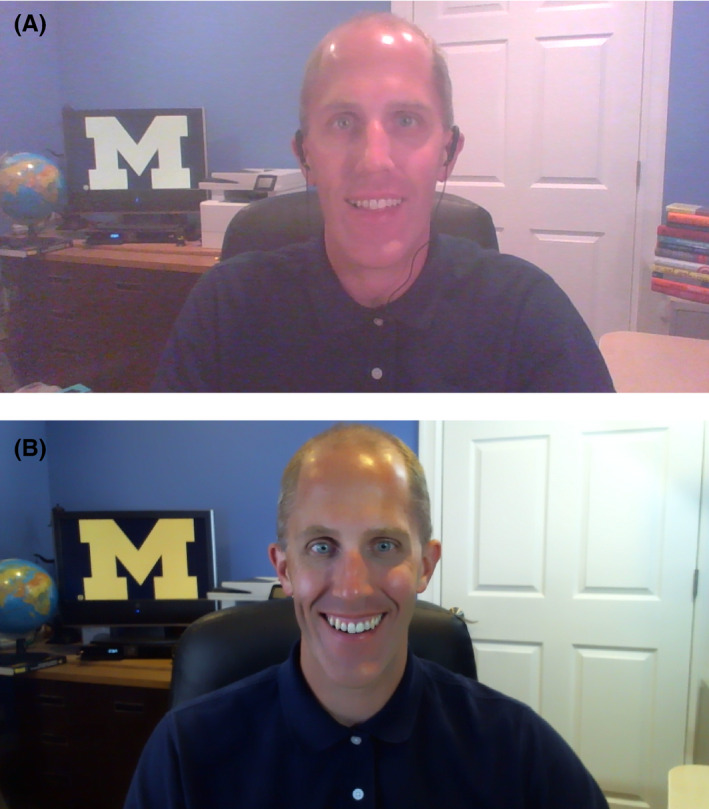
Camera and lighting these two pictures illustrate the difference between standard and upgraded camera and lighting configuration. Configuration (A) leverages standard video camera and lighting while (B) leverages upgraded camera and three point lighting configuration. Either is perfectly acceptable, but configuration (B) provides greater clarity and professionalism

**FIGURE 2:**
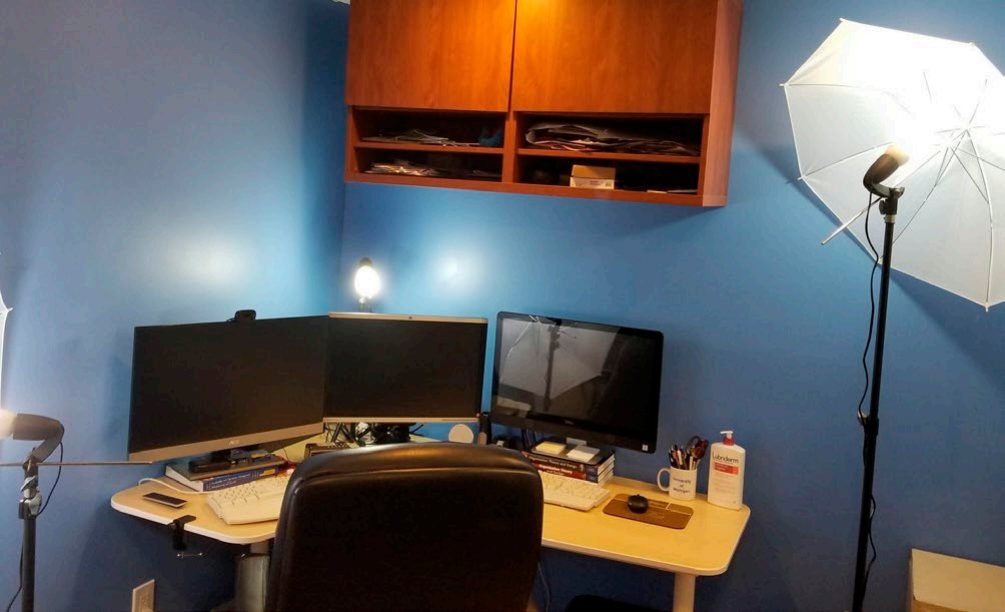
Behind the scenes setup. Where the previous figure showed the student view, this is my view, including the setup of three point lighting.

The basic camera and microphone in most computers is generally sufficient for teaching. However, upgrading to a higher quality microphone can make you even clearer to hear and give your presence a more “professional” ambiance. Additionally, a higher quality camera can result in clearer and crisper video quality. Another consideration is lighting. Even the highest quality camera cannot compensate for poor lighting. Your face should be lit from the front to clearly show your facial features and expressions. Also, you should avoid having a direct source of light behind you (for example, don’t sit in front of a window), as it will cast you in shadow and make you hard to see. For advanced lighting configuration, consider a classic three point lighting set up which places one light in front of you on either side and a third light illuminating you from behind. The combined impact of the three lights is to minimize shadows and maximize a natural, well‐lit appearance. There are many online tutorial videos that elaborate on this technique. Figure 1 shows the difference between standard and upgraded lighting and camera configurations, and Figure 2 shows the entire setup from behind.

Some video platforms also provide the option to use a virtual background, that is, replacing your natural video background with an image from your computer. While technology of yesteryear required advanced lighting and backgrounds (eg, a “green screen”), modern technology no longer has those requirements, making it accessible to both us and our students. However, just because it is accessible, doesn’t mean we need to use it. This is somewhat a matter of personal choice: what message or impression do you want to convey to your students? Does your natural background convey that, or would you rather have another background? Additionally, while the technology is much‐improved, virtual backgrounds can sometimes be distracting as the software is still limited in its accuracy of determining the boundary between someone’s face and the background, and may not display a portion of the face, or may display a portion of the background. Virtual backgrounds can also be used to provide variety or help augment the context of a given lecture (for example, I once used a factory background on a unit involving manufacturing). However, to be very clear: virtual background or not, the lighting concerns discussed in the previous paragraphs still apply. Even when using a virtual background, good lighting is still needed for your personal appearance.

The technology described in this section alone will not ensure a great online learning experience for students. Rather, these serve to help minimize the distraction technology could otherwise be so that students can focus on learning.

## 8 GROUP WORK

My courses involve a significant amount of group work. One of the most surprising aspects of the transition to online learning for me was the difficulty many students felt in completing group work when they weren’t together physically. Understanding how to work together for group work was easily the most frequent process‐related question I received about the transition from in‐person to online learning. I realized an important lesson in this: as I was adapting to our new online realities, the students were too. They needed greater guidance and structure around how to think about interacting with group members and completing assignments together virtually.^15^

I recommend explicitly taking class time to discuss how to work effectively in groups virtually. Leverage the wisdom of the room and ask for students to share their own ideas of what has and hasn’t worked for them. Students need to develop self‐efficacy in effective online learning and group work behaviors.^16^ The discussion should in some ways parallel themes in this article ‐ setting common expectations, having synchronous meetings for at least some of the work, and understanding and leveraging technical tools to enable their collaboration. In particular, on setting common expectations, expectations of communication are especially important. Each group needs to establish their expected shared methods of communicating, whether email, group text, or other platform, as well as a high level timeline of milestones and expectations in working together.

## 9 CULTURE AND EXPECTATIONS

As instructors, we set a classroom culture beginning with our first interaction with students. This culture includes what is “normal” or expected in our classroom, how students interact with the professor, how students interact with each other, and the entire classroom experience. We set this culture whether we are intentional about it or not. In a virtual environment, especially with the relative newness of the virtual environment for many of our students, it is important that we are intentional in setting the culture we desire, and in reinforcing that throughout the semester through explicit reminders as well as more implicit recognizing and encouraging of the types of behaviors we want to see. Organizations such as Quality Matters^17^ provide comprehensive guidance for faculty and institutions in consideration what expectations to set and how in order to deliver high‐quality online learning.

At a foundational level, this includes expectations for the course as clearly outlined in the syllabus. It now includes the added layer of using all of the technology we’ve discussed, from having video turned on, to using the hand raise function, to what to write in chat, to how to use annotation, to how often to take breaks to avoid fatigue from looking too long at their computer screens. Additionally, setting expectations and building culture includes how students are expected to carry themselves in class and the amount of interaction and contribution. In my case, I begin this conversation with a simple question prompt: *think of your best class experience ever, what made it such a great experience?* Inevitably, this draws out answers related to how interactive the class is, and how connected students feel to their peers. Further, this helps draw out differences in learning styles and how to best accommodate those styles.^18^ Exploring with students what they then want that interaction to look like in your classroom helps establish a great foundation for an expected culture of engagement. Table 2 summarizes overall recommendations for establishing an effective online learning environment.

**TABLE 2:** Recommendations for effective online learning

Host synchronous and interactive classes to enable discussion and synthesize of materials Establish clear norms and expectations for the online environment in order to guide student behaviors to maximize their learning and minimize unmet expectations Use interactive video conferencing features (sharing video, hand raising, small group discussions, chat, etc) to engage students Help students through the transition to virtual learning, such as by providing process guidance for group work Display empathy and flexibility to student pressures and circumstances at this especially difficult time Don’t limit yourself to what was possible in physical classrooms, strive to enhance the experience beyond what is possible in‐person

## 10 LOOKING AHEAD

We are in a time of experimentation. There are many articles around the experiences of individuals and institutions, what has worked, and what has not worked in the transition to remote learning. Just as we each bring our experiences and personalities to our classrooms in a way that creates our unique brand of experience for students, so too in the online environment. However, where the online environment perhaps diverges dramatically from the in‐person environment is the volume and configurability of the tools available to us. A goal of this article was to provide thoughtful exposure to some of those key tools so that you can more fully consider how they may integrate, or not, with your own teaching philosophies and approach.

Until a COVID‐19 vaccine is developed and administered, we will likely continue to live with at least some forms of social distancing. As we consider this short‐term reality, envision the socially‐distanced in‐person classroom. This involves a much larger classroom for a comparable number of students, so that we can maintain safe levels of physical distancing. This also involves wearing masks. Being spaced far apart and wearing masks. Envision small group discussions in this sort of setting. Envision trying to read non‐verbal cues, or even being able to know if someone is talking by looking at them. I can’t help but think the online learning environment in some ways enables a better learning environment for larger classes, where students are not wearing masks while online and where our cameras provide a clear view of each other. Perhaps a combination of in‐person and virtual should be considered in the interim. Systematic literature review suggests a blended approach including in‐person and online components may afford the balance of benefits needed.^19^

Many of us, myself included, having a longing to return to “the way it was.” However, we are in our new COVID‐19 pandemic reality and will be here for some time. Rather than resist this reality, and try to go back to “the way it was”, how can we dive‐in and truly make the most of online learning for our students? Instead of striving only to make online learning as good as in‐person instruction, what if we used this as an opportunity to make it better? I’m convinced that the opportunity of the present time is not only related to online learning, but that our designing for interactive online learning can also directly translate into improving in‐person interactive learning into the future. The experiences and insights in this article are not meant to be limited only to improving online learning, but are focused on improving learning for our students regardless of in‐person or virtual instruction. I hope my experiences and reflections have provided you with at least some form of inspiration or insight.

## CONFLICT OF INTEREST

The author has no conflict of interest to declare.
